# Genomic selection applications can improve the environmental performance of aquatics: A case study on the heat tolerance of abalone

**DOI:** 10.1111/eva.13388

**Published:** 2022-05-13

**Authors:** Junyu Liu, Wenzhu Peng, Feng Yu, Yawei Shen, Wenchao Yu, Yisha Lu, Weihong Lin, Muzhi Zhou, Zekun Huang, Xuan Luo, Weiwei You, Caihuan Ke

**Affiliations:** ^1^ 12466 State Key Laboratory of Marine Environmental Science College of Ocean and Earth Sciences Xiamen University Xiamen China; ^2^ 12466 Fujian Key Laboratory of Genetics and Breeding of Marine Organisms Xiamen University Xiamen China

**Keywords:** abalone, climate change, genome‐wide association study, genomic selection, heat tolerance

## Abstract

Aquaculture is one of the world's fastest‐growing and most traded food industries, but it is under the threat of climate‐related risks represented by global warming, marine heatwave (MHW) events, ocean acidification, and deoxygenation. For the sustainable development of aquaculture, selective breeding may be a viable method to obtain aquatic economic species with greater tolerance to environmental stressors. In this study, we estimated the heritability of heat tolerance trait of Pacific abalone *Haliotis discus hannai*, performed genome‐wide association studies (GWAS) analysis for heat tolerance to detect single nucleotide polymorphisms (SNPs) and candidate genes, and assessed the potential of genomic selection (GS) in the breeding of abalone industry. A total of 1120 individuals were phenotyped for their heat tolerance and genotyped with 64,788 quality‐controlled SNPs. The heritability of heat tolerance was moderate (0.35–0.42) and the predictive accuracy estimated using BayesB (0.55 ± 0.05) was higher than that using GBLUP (0.40 ± 0.01). A total of 11 genome‐wide significant SNPs and 2 suggestive SNPs were associated with heat tolerance of abalone, and 13 candidate genes were identified, including *got2*,*znfx1*,*l(2)efl*, and *lrp5*. Based on GWAS results, the prediction accuracy using the top 5K SNPs was higher than that using randomly selected SNPs and higher than that using all SNPs. These results suggest that GS is an efficient approach for improving the heat tolerance of abalone and pave the way for abalone selecting breeding programs in rapidly changing oceans.

## 1 INTRODUCTION

With the potential of mitigating negative impacts associated with land‐based food production systems, aquaculture is one of the world's fastest‐growing and most traded food industries, with Asia accounting for 90% of worldwide production (FAO, 2018; Stentiford et al., 2020). Aquatic foods from marine and freshwater systems present an opportunity to improve nutrition with lower environment burdens and sustainably development (Gephart et al., 2021; Parker et al., 2018). Global demand for aquatic foods has roughly doubled since the turn of the 21st Century and will likely double again by 2050 (Naylor et al., 2021). However, climate‐related risks, including global warming, marine heatwave (MHW) events, ocean acidification, and deoxygenation, may jeopardize the ability of aquaculture to deliver these services, as well as sustainable food system outcomes (Tigchelaar et al., 2021). Among these risks, MHW events (Hobday et al., 2016) have increased in frequency all around the world, causing severe impacts on marine ecosystems and threatens all aspects of aquatic food system, from production to consumption (Barange et al., 2018; Caputi et al., 2016; Myers et al., 2017; Perkins et al., 2012; Smale et al., 2019).

Marine sessile and adhering organisms are among the most vulnerable to heatwaves because they cannot physically remove themselves and seek refuge. As the representative species, abalone is an economically important resource but suffered mass mortalities and disease outbreaks by heat stress all around the world, such as green abalone (*Haliotis fulgens*; Calderón‐Liévanos et al., 2021; Samuel et al., 2019), red abalone (*Haliotis rufescens*; Hart et al., 2020), black abalone (*Haliotis cracherodii*; Ben‐Horin et al., 2013), black‐lip abalone (*Haliotis rubra*), and green‐lip abalone (*Haliotis laevigata*; Roberts et al., 2019; Shiel et al., 2020). The Pacific abalone, *Haliotis discus hannai*, is the main aquaculture abalone species in China (Li et al., 2012; Nguyen et al., 2013), which has been the biggest abalone production area in the world and produced 89% of the total aquaculture abalone production (FAO, 2021). With the highest seawater temperatures nearing 30°C, the abalone industry in southern China is suffering severe summer mortality events (Cheng et al., 2004; Dang et al., 2012; Kyeong et al., 2020; Shen et al., 2019). For the sustainable development of aquaculture, selective breeding may be a viable method to obtain aquatic species with greater heat tolerance.

In the last decade or two, highly efficient, cutting‐edge molecular tools have been developed to aid modern breeding programs, including genome‐wide association study (GWAS) and genomic selection (GS). GWAS is a powerful tool for connecting phenotypes of organisms to genetics and for investigating the molecular mechanisms underlying specific phenotypes (Korte & Farlow, 2013). Genomic selection (GS; Meuwissen et al., 2001) is a form of molecular marker‐assisted selection, which utilizes high‐dense molecular genetic markers throughout the whole genome to estimate the breeding value, namely genomic estimated breeding value (GEBV), of candidates. Compared with mass selection and traditional selection methods, GS has obvious advantages, especially for the traits with low heritability and difficult to measure (Meuwissen et al., 2013; Xu et al., 2021). With the fast development of SNP genotyping techniques (Bhat et al., 2016; Tan et al., 2017), the applications of GS for growth, disease resistance, and quality traits have been documented in many aquatic species, such as rainbow trout (Vallejo et al., 2019), tilapia (Lu et al., 2020), shrimp (Liu et al., 2020; Wang et al., 2019), and Yesso scallop (Dou et al., 2016). However, the applicative potential of GS for trait improvement of the Pacific abalone has not been determined.

In this study, a total of 1120 individuals of Pacific abalone *H*. *discus hannai* were phenotyped for their heat tolerance and genotyped with whole‐genome resequencing. Then, we estimated the heritability of heat tolerance trait of Pacific abalone, performed GWAS analysis for heat tolerance to detect single nucleotide polymorphisms (SNPs) and candidate genes, and evaluated the potential of GS in the breeding of abalone industry. Our results have guiding significance for the heat tolerance breeding of marine economic species to cope with the climate changes and food problems around world.

## 2 MATERIALS AND METHODS

### 2.1 Sample collection and assessment of heat tolerance

The Pacific abalones used in this study were from a commercial breeding company (Fuda Abalone Farm, Jinjiang, China). A total of 1121 individuals were randomly selected in the population constructed from 400 broodstocks with a random mating design.

Seventeen‐month‐old abalone (body weight: 25.23 ± 5.96 g, mean ± SD) were labeled and acclimated at 20°C for 2 months. A modified and more efficient method based on method published by Yu, Wu, et al. (2021) was used to assess the heat tolerance of the abalone individuals. Abalones were placed into four thermostatic recirculation systems each with four glass tanks (50 cm × 45 cm × 30 cm for each glass tank). The seawater temperature in the circulating systems was increased at a rate of 1°C/h and maintained at 32°C for 6 h, and adhesion substrates where abalones attach suspended horizontally underwater. Under continuous extreme heat stress, abalones gradually lose adhesion and detach. The attachment durations of individuals in this study were recorded and they were allowed to recover in fresh flowing seawater at 20°C.

### 2.2 Individual genotyping

The foot muscle of 1121 tested individuals was collected and stored at −80°C for DNA extraction. Genomic DNA was extracted using the DNeasy 96 Blood & Tissue Kit (Qiagen) and quantified by Nanodrop2000 (Thermo Scientific). Sample genotyping was carried out by MOL BREEDING (Hebei, China) using a 40K multiple single nucleotide polymorphism (mSNP) array and 87,959 SNPs were obtained. Quality control of sequencing data was performed using PLINK software (Purcell et al., 2007), with the criteria of minor allele frequency (MAF) >0.05, SNP call rate >0.95, and individual genotype call rate >0.8. A dataset of 1120 abalones with genotypes of 64,788 SNPs remained after quality control, and the average missing rate of markers was 0.8%. All missing SNPs were imputed with software BEAGLE v4.0 (Browning & Browning, 2007). The default parameter settings for Beagle were used, except for setting the effective population size to the number of individuals of 1120 instead of the default of 1 million for human (Browning & Browning, 2016; Wu et al., 2019).

### 2.3 Genome‐wide association study (GWAS) and candidate gene annotation

Population structures were first examined to estimate the potential genetic relatedness within population using principal components analysis (PCA) in PLINK before association analysis (Figure S1). Then, each principal component was tested for significance based on Tracy–Widom statistics in EIGENSOFT (Li, Qian, et al., 2012; Patterson et al., 2006). GWAS were performed using a Bayesian information and Linkage‐Disequilibrium Iteratively Nested Keyway (BLINK; Huang et al., 2019) by GAPIT (Wang & Zhang, 2020). The models included the first three significant principal component factors (*p* < 0.05) and a kinship matrix as fixed and random effects, allowing control of the effects caused by population stratification and relatedness (Rincker et al., 2016). The Manhattan plot of −log_10_(*p* value) and the QQ plot of the original significance level (*p* value) were produced by the *qqman* package (Turner, 2014) in R. The *p* value for genome‐wide significance was set as 0.05/64,788 = 7.72e‐07 (−log_10_(*p* value) = 6.11) based on the Bonferroni test (Bonferroni, 1936; Goeman & Solari, 2014). The suggestive significance was arbitrarily set as the conventional threshold of 1e‐5 (−log_10_(*p* value) = 5) (Coltell et al., 2020; Yu, Peng, et al., 2021). To annotate the candidate genes, genes located on or adjacent to the SNPs exceeding suggestive threshold were identified as candidate genes associated with thermal resistance.

### 2.4 Genetic parameter estimation

The variance components for HAD were evaluated using *ASReml*‐*R* (Butler et al., 2009) and *BGLR* packages (Pérez & de Los Campos, 2014) in R. The GBLUP model can be described by the following equation:

(1) y=Xb+Zg+e,

where *y* is the phenotype vector; *b* is the fixed effects vector containing two factors (intercept and different batches); *g* is the random effects vector, which is assumed to follow ~*N* (0, *G* $\sigma_{g}^{2}$), where G is the genomic relationship matrix (VanRaden, 2008), $\sigma_{g}^{2}$ is the additive genetic variance; *X* and *Z* are the corresponding incidence matrices for fixed and random effects, respectively; and *e* is a vector of the random residual effects ~*N* (0, *I* $\sigma_{e}^{2}$), where I is the identity matrix and $\sigma_{e}^{2}$ is the residual variance. The phenotype variance ($\sigma_{P}^{2}$) for HAD was calculated as: $\sigma_{P}^{2}=\sigma_{g}^{2}+\sigma_{e}^{2}$, and the narrow‐sense heritability was computed as the ratio between additive genetic variance and phenotype variance: $h^{2}=\sigma_{g}^{2}/\sigma_{P}^{2}$.

BayesB is a mixed model, in which a priori SNP effect is assumed to be zero with probability *π*, and normally distributed with a mean equal to 0 and a locus‐specific variance with probability (1 − *π*). The Bayes B model is the following:

(2) y=Xb+Mu+e,

where *y*,*Xb*, and *e* are the same as terms described in Equation (1); *M* is the matrix of genotypes encoded as 0, 1, and 2 copies of the reference allele; and *u* is the sum of SNP effects that are derived from two independent distributions:

$$p\left(u|\pi ,\sigma_{u}^{2}\right)=\left\{\begin{array}{cc} 0, & \text{with}\;\text{probability}\;\pi \\ ∼N\left(0,\sigma_{u}^{2}\right), & \text{with}\;\text{probability}\;(1‐\pi ) \end{array}\right.,$$

where $\sigma_{u}^{2}$ is the additive genetic variances explained by SNPs and obey an inverse Chi‐square distribution, *π* is set to 0.95, and the unknown parameters (*b*,*u*, $\sigma_{u}^{2}$) are obtained from a Gibbs scheme‐based Monte Carlo Markov Chain (MCMC) iterations. A total of 100,000 iterations were used in the Gibbs sampling, and the first 3000 iterations were discarded as burn‐in cycles.

### 2.5 Calculation of prediction accuracy

The fivefold cross‐validation (CV) was used to compare the prediction accuracy of GBLUP and BayesB. The 1120 individuals were randomly separated into five groups of approximately equal size. Each group was used only once as a testing set in five analyses. For each analysis, the phenotypes of the testing set were set as “missing” and the remaining phenotypes were used as the training set. For the two methods, prediction accuracy was calculated as the Pearson correlation between phenotypes and GEBVs. The fivefold CV was repeated 10 times for each CV scenario, and the average values were calculated.

The bias of the GEBV was calculated as the regression coefficient of observed phenotypes on GEBV. A regression coefficient of <1 or >1 indicates that GEBV is an overestimation or underestimation of the breeding value (BV). Therefore, a significant deviation from 1.0 is usually interpreted as prediction bias.

### 2.6 Selection of SNPs number in the GS model

For investigating the appropriate numbers of SNP for the two GS models, two SNP panels based on the *p* values of GWAS in ascending order and randomly selected markers were developed. Datasets containing different numbers of SNPs were generated: 500, 1000, 5000, 10,000, 20,000, 40,000, and all SNPs. For each SNP subset, the GS model was built, and corresponding predictability was calculated through fivefold CV as described above.

## 3 RESULTS

### 3.1 Phenotype statistics

**FIGURE 1:**
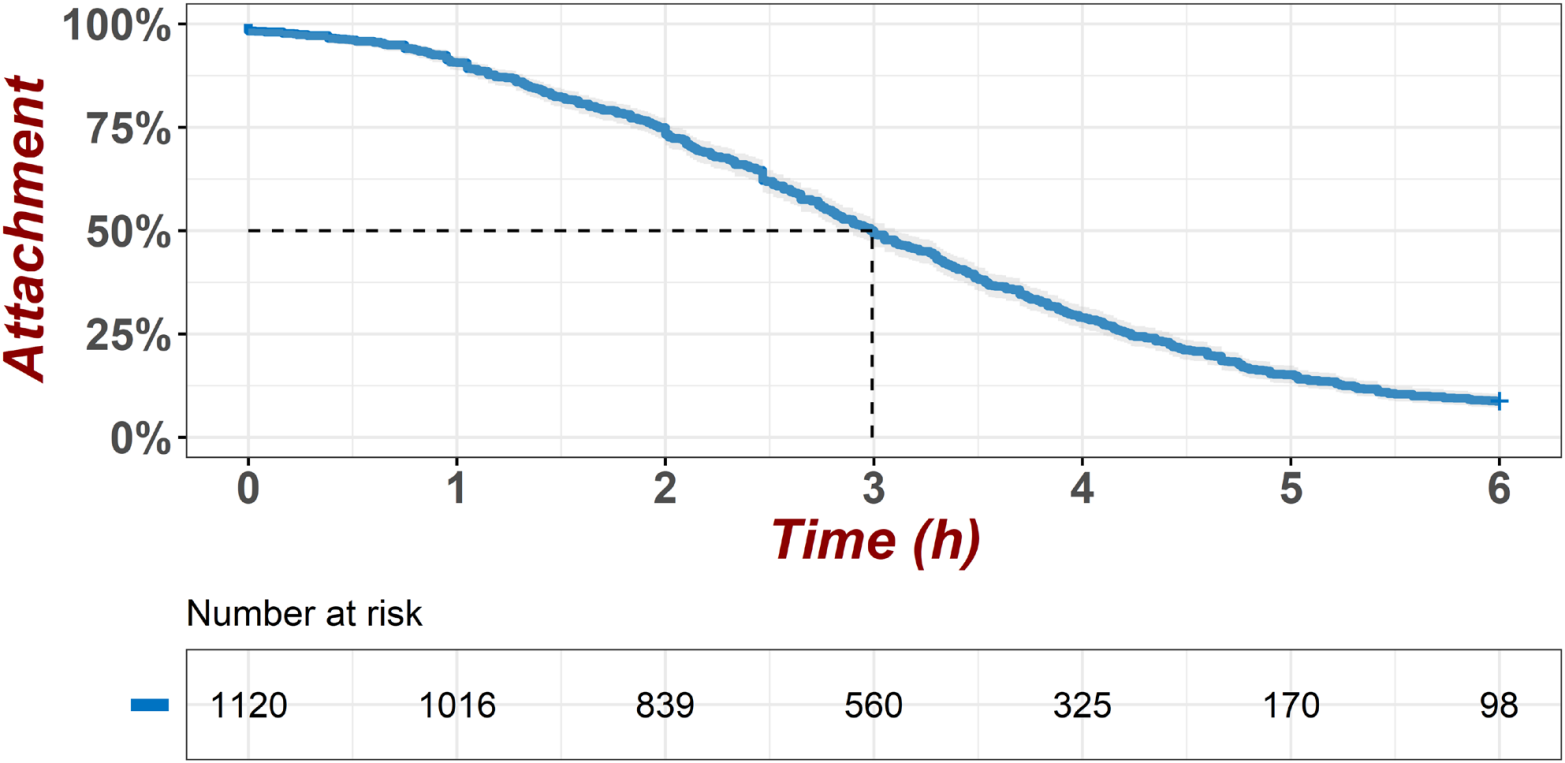
Attachment curves of abalones under 32°C heat exposure

The attachment curves of abalones under 32°C heat exposure are shown in Figure 1. The drop rate (24.91%) peaked at the 3^rd^ h and the cumulative drop rate reached 50% at the same time. The heat exposure lasted for 6 h and the final attachment rate, at the end of the 6‐h exposure, was 8.75%.

### 3.2 GWAS and putative genes identification

**FIGURE 2:**
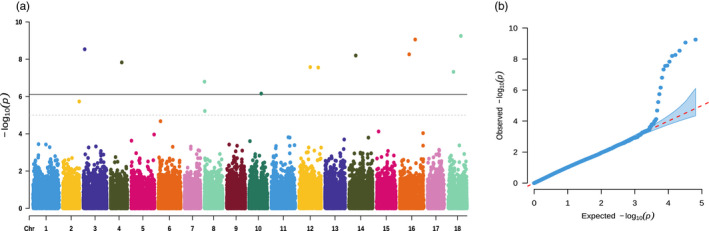
(a) Manhattan and (b) quantile–quantile (QQ) plots visualizing the results of GWAS for heat tolerance in the Pacific abalone. In the Manhattan plot, the gray dotted line represents the suggestive threshold (−log^10^(1 × 10^−5^) = 5) and the black line represents the genome‐wide significance threshold (−log^10^(7.72 × 10^−7^) = 6.11)

Eleven genome‐wide significant SNPs and two suggestive SNPs were found (Figure 2 and Table 1). These SNPs were located in multiple linkage groups, and the most significant loci was LC47339815 on chromosome 18 (*p* value = 5.62e‐10; Table 1), which explained 1.04% of the phenotype variance. The minor allele frequencies of these variants ranged from 0.05 for SNP LC70081983 to 0.45 for SNP LC20163442. The phenotypic variance explained (PVE) by the top markers ranged from 0.34% to 1.04%.

**TABLE 1:** Details of SNPs associated with heat tolerance in Pacific abalone

SNP	Chr.	Position	Region	Allele	MAF	*p* Value	PVE
LC47339815	18	47339815	Intergenic	G/A	0.11	5.62E‐10	1.04
LC57373210	16	57373210	Intron	A/G	0.12	8.75E‐10	0.79
LC5223508	3	5223508	Intergenic	A/C	0.08	2.92E‐09	0.37
LC35299997	16	35299997	Intron	G/A	0.1	5.45E‐09	0.98
LC25289967	14	25289967	Intergenic	G/A	0.17	6.33E‐09	0.6
LC41179727	4	41179727	Intron	A/T	0.08	1.48E‐08	0.89
LC40643987	12	40643987	Intergenic	A/G	0.16	2.64E‐08	0.34
LC70081983	12	70081983	Intron	T/A	0.05	2.78E‐08	0.36
LC20163442	18	20163442	Intergenic	A/C	0.45	4.74E‐08	0.21
LC1059454	8	1059454	Intergenic	G/A	0.11	1.60E‐07	0.99
LC44890630	10	44890630	Intron	T/A	0.11	6.98E‐07	0.53
LC60474408^a^	2	60474408	Intergenic	C/A	0.22	1.84E‐06	0.51
LC2286791^a^	8	2286791	Intron	G/A	0.09	6.00E‐06	0.61

Abbreviations: Allele, minor/major allele; Chr., chromosome; MAF, minor allele frequency; PVE, phenotypic variance explained.

Suggestive SNP.

After mapping onto the reference genome (unpublished), a total of 13 genes were identified across all 13 SNPs exceeding suggestive threshold (Table 2), including lethal (2) essential for life (*l(2)efl*), aspartate aminotransferase (*got2*), transmembrane domain protein (*ptch1*) and zinc finger protein family (*znfx1*). The candidate genes serve essential functions in multiple biological processes such as in transmembrane, metabolic pathways.

**TABLE 2:** Candidate genes identified in the GWAS analysis

Gene ID	Chr.	Location (bp)	Gene name	Gene annotation
HDH_T04192	2	60370525–60371226	*map1a*	Microtubule‐associated protein 1A
HDH_T07343	4	41167930–41191594	*l(2)efl*	Protein lethal (2) essential for life
HDH_T12180	8	2282783–2287958	*cg11103*	TM2 domain‐containing protein CG11103
HDH_T16270	10	44887481–44906319	*slx1a*	Structure‐specific endonuclease subunit slx1
HDH_T19811	12	40640155–40642539	*foxa2*	Hepatocyte nuclear factor 3‐beta
HDH_T20589	12	70074545–70084599	*lrp5*	Low‐density lipoprotein receptor‐related protein 5
HDH_T23216	14	25219669–25274748	*thop1*	Thimet oligopeptidase
HDH_T23217	14	25295287–25359182	*ptch1*	Protein patched homolog 1
HDH_T27723	16	57369261–57412231	*gyc32e*	Guanylate cyclase 32E
HDH_T27153	16	35299484–35304267	*pbld*	Phenazine biosynthesis‐like domain‐containing protein
HDH_T30820	18	47303881–47312003	*znfx1*	NFX1‐type zinc finger‐containing protein 1
HDH_T30821	18	47369312–47440511	*g13g3.3*	UPF0392 protein F13G3.3
HDH_T30141	18	20173749–20219231	*got2*	Aspartate aminotransferase

### 3.3 Variance components and heritability

The variance components and heritability for HAD estimated using GBLUP and BayesB are given in Table 3. The additive genetic variances of GBLUP (0.92 ± 0.17) and BayesB (0.89 ± 0.09) were similar, and the heritability of HAD, calculated by GBLUP (0.42) and BayesB (0.35), is in the moderate heritability category.

**TABLE 3:** Variance components and heritability values of heat tolerance‐related trait estimated using different methods in Pacific abalone

Method	$\sigma_{a}^{2}$	$\sigma_{e}^{2}$	$\sigma_{p}^{2}$	*h* ^2^
GBLUP	0.92 ± 0.17	1.26 ± 0.10	2.18 ± 0.12	0.42 ± 0.06
BayesB	0.89 ± 0.10	1.65 ± 0.08	2.54 ± 0.10	0.35 ± 0.03

### 3.4 Predictability accuracy estimation

The predictive performance of GBLUP and BayesB methods with a fivefold cross‐validation was determined. The prediction accuracy of BayesB was 0.55 ± 0.05 which was higher than GBLUP (0.40 ± 0.01). The regression coefficients of GEBV on phenotypes were 0.98 ± 0.08 and 1.04 ± 0.02 for BayesB and GBLUP. The predictions obtained by both methods were similar.

### 3.5 Prediction performance of different SNP panels

**FIGURE 3:**
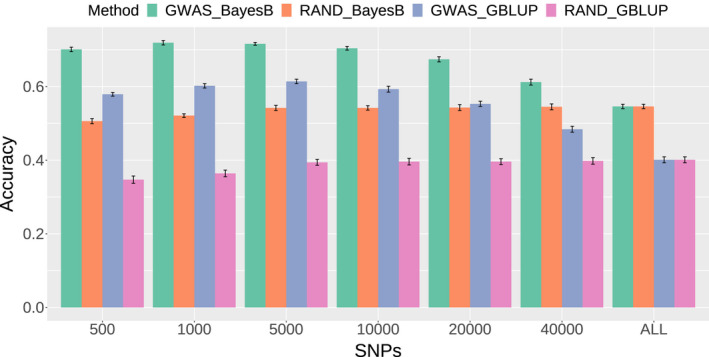
Genomic prediction accuracy for heat tolerance‐related trait using different methods and different numbers of SNPs. The green and blue columns represent the prediction accuracy based on SNPs selected by GWAS using the BayesB and GBLUP methods, respectively. The orange and red columns represent the prediction accuracy based on randomly selected SNPs using BayesB and GBLUP models, respectively

Based on the GWAS results, the top 500, 1K, 5K, 10K, 20K, and 40K SNPs were selected as subset data for genomic prediction, and randomly selected SNPs were also performed for comparisons. The prediction accuracy using the top SNPs selected was higher than that of randomly selected SNPs and the accuracy predicted by the BayesB method was higher than the GBLUP method (Figure 3). For the top SNPs, the highest prediction accuracy was achieved by using GBLUP (0.61 ± 0.01) and BayesB (0.72 ± 0.01) when the number of SNPs reached 5000. The prediction accuracy achieved by using 5000 top SNPs was increased by 30.91% (GBLUP) and 52.50% (BayesB) compared to using all SNPs. For the randomly selected SNPs, the prediction accuracy was improved as marker density increased and 5K SNPs showed similar prediction accuracy to 64,788 SNPs.

## 4 DISCUSSION

Marine and atmospheric heatwaves are becoming more frequent across the globe, and they are causing substantial losses to the aquaculture industry. Initiatives to build the resistance of marine organisms to heatwaves have accelerated in recent years. Researchers are taking a series of selective breeding measures to improve the tolerance of wild coral to bleaching caused by heatwaves (Van Oppen et al., 2015). Similar strategies are also being used to improve the tolerance of macroalgae to heatwaves (Layton et al., 2020). In aquaculture, some species are selectively bred to improve their resistance to climate change stress. Selectively bred populations of oyster *Saccostrea glomerata* had greater extracellular pH when exposed to elevated pCO_2_ compared to wild oysters, and were resistant to ocean acidification (Parker et al., 2012, 2015). The study of Atlantic salmon (*Salmo salar*) highlighted that selective breeding programs and other more advanced biotechnological solutions (e.g., gene editing) may play a key role to cope with a changing ocean (Calado et al., 2021). In this study, we estimated the heritability of heat tolerance of abalone and found that it was moderate. At the same time, the performance of genomic selection was assessed; the results showed that GS is an efficient selection breeding tool for heat tolerance in Pacific abalone. Based on sorted GEBV values calculated using phenotype and genotype data, candidates with better resistance to heat stress were selected as broodstocks. Utilizing the GS method can accelerate selection process and improve genetic gains for heat tolerance of abalone.

Narrow‐sense heritability is a central parameter of quantitative genetics and has a predictive role in aquaculture breeding. The estimated heritability for heat tolerance of the Pacific abalone ranged from 0.35 to 0.42, which had a moderate degree of heritability. For a given heat tolerance trait, the estimated heritability varied in previous studies. In rainbow trout (*Oncorhynchus mykiss*), the heritability for heat tolerance‐related traits ranged from 0.41 to 0.51 (Ihssen, 1986; Perry et al., 2005), but relatively lower heritability values were observed in Pacific oyster *Crassostrea gigas* (0.15; Camara et al., 2017) and turbot *Scophthalmus maximus* (0.026; Liu et al., 2011). Estimates were influenced by many factors such as species, statistic model and specific trait assessment method in the analysis. In a study on *P*. *salmonis* resistance in Atlantic salmon, heritability was estimated to be 0.18 when the resistant trait was defined as the day of death, and 0.24 when the resistant trait was treated as a binary variable, survival or mortality (Yáñez et al., 2013). Estimated heritability was varied using two statistic models (ssGBLUP: 0.33 vs. BayesB: 0.23) when investigating the bacterial cold water disease resistance in rainbow trout (Vallejo et al., 2017). The heritability of heat tolerance in the Pacific abalone suggests that the genetic gain can be enhanced through selective breeding.

When performed GS analysis with different model, we found that the predictive accuracy estimated using BayesB (0.55 ± 0.05) was higher than that using GBLUP (0.40 ± 0.01). The difference between GBLUP and BayesB was largely dependent on the model of genetic variation used to simulate the underlying variation. Bayesian models often have an advantage over the GBLUP model when the architecture of the trait is (partly or entirely) controlled by a number of major quantitative trait loci (QTLs). However, Clark et al. (2011) reported that, as the number of QTLs increased (the variation of the largest QTL explained less) the ability of BayesB to predict breeding values decreased (Clark et al., 2011). Daetwyler et al. (2010) obtained similar results when they investigated the impacts of genetic architecture on genome‐wide evaluation methods (Daetwyler et al., 2010). In the present study, the predictive accuracy of the BayesB method was slightly higher than GBLUP (Figure 3), and there was no significant difference in the prediction of heat tolerance‐related traits, suggesting that the trait may be controlled by several minor QTLs, which is consistent with the result of GWAS.

In addition to predictive accuracy, cost‐efficiency is important for the practical use of GS for aquatic animals with lower individual economic value. Selecting an optimal SNP panel can improve the accuracy of genomic selection and decrease the genotyping costs. The prediction accuracy estimated by GBLUP and BayesB using the top 5000 SNPs was higher than those using randomly selected SNPs, and higher than those using all 64,788 SNPs (Figure 3). Therefore, a relatively lower density of SNP panel selected by GWAS can be used in GS breeding of abalones. This might be due to the fact that most trait‐associated SNPs, which can explain most of the genetic variation, were identified by GWAS. Similar results were found in the orange‐spotted grouper (Shan et al., 2021), shrimp, salmon, and gilthead seabream (Luo et al., 2021). In studies on *Oplegnathus fasciatus*, the predictive abilities using informative SNPs sorted by GWAS showed a more stable upward trend compared with the predictability estimated by randomly sampled SNPs from the genome as the SNP numbers increased (Gong et al., 2021).

It is significant to explore and understand the mechanisms of thermal tolerance of Pacific abalone. GWAS is an efficient way to elucidate the genetic variations associated with complex quantitative traits and to yield candidate genes and markers for mechanism research and molecular‐assisted breeding. Thirty candidate genes were predicted in the study of large yellow croaker on acute heat tolerance (AHT) and provided insights into the genetic basis of AHT in fish and will be helpful for heat‐tolerance improvement of large yellow croaker (Wu et al., 2021). Similar study were reported in catfish, a total of 14 genes with heat stress‐related functions were detected within the significant associated regions (Jin et al., 2017). In our study, we identified 13 SNPs associated with the heat tolerance of abalones, and they explained 8.22% of the phenotypic variance. These SNPs were located on multiple chromosomes and did not show obvious clustering. The heat tolerance‐related trait in abalones is likely complex and controlled by multiple genes with minor effects. This would be consistent with the heat tolerance of the large yellow croaker (Wu et al., 2021) and Pacific oyster (Meng et al., 2020).

A total of 13 candidate genes associated with the heat tolerance of abalone were identified. Among them, several related genes serve essential functions in multiple biological processes such as in transmembrane (May et al., 2007), and metabolic pathways (Arun et al., 2013) are worthy of our attention. Aspartate aminotransferase (got2) plays a critical role in metabolism and energy production (Arun et al., 2013). Ekström et al. (2017) investigated the effects of temperature increase on the thermal sensitivity of 10 key enzymes governing cardiac oxidative and glycolytic metabolism in *Perca fluviatilis* and suggested that the malate–aspartate shuttle may help to maintain cardiac oxidative capacity at high temperatures (Ekström et al., 2017). The NFX1‐type zinc finger proteins (*znfx1*) belong to a group of human NFX1 transcription factors that may act as transcriptional repressors in regulating the duration of inflammatory responses (Song et al., 1994). The *znfx1* was upregulated during salinity stress in *Crassostrea gigas* (Zhao et al., 2012) and under nervous necrosis virus persistent infection in *Epinephelus malabaricus* (Tso & Lu, 2018). Lethal (2) essential for life (*l(2)efl*) and the low‐density lipoprotein receptor (*lrp5*) also play important roles in the maintenance of protein homeostasis and transport of nutrients and vitamins (Jagla et al., 2018; Landis et al., 2012; Lu et al., 2014; May et al., 2007). The related genes identified in this study are evidence of their relationship with heat tolerance and help to clarify the genetic mechanisms underlying abalone heat tolerance.

In conclusion, ocean warming and marine heatwave events can be lethal to the survival of abalones in the aquaculture areas and cause ecological and economic losses to global aquaculture. However, it is a feasible way to explore the potential to augment the capacity of abalone to tolerate stress in an era of unprecedented climate change and food shortage problems around the world. The application of modern cutting‐edge molecular tools allows us to efficiently obtain abalone species/strains with heat tolerance. In this study, our results found that GS is an effective approach and can accelerate selective breeding process for heat tolerance of Pacific abalone. A total of 13 markers and 13 candidate genes were identified to be associated with heat tolerance of Pacific abalone. Overall, these molecular markers would further help to clarify the genetic architecture and molecular mechanisms of heat tolerance in abalone and support the development of marker‐assistant selection in abalone breeding programs.

## CONFLICT OF INTEREST

The authors declare that they have no conflict of interest.

## Supporting information

Fig S1

## Data Availability

The data that support the findings of this study are available from the corresponding author upon reasonable request.
