# Supplementary figures and images for: Genomic selection applications can improve the environmental performance of aquatics: A case study on the heat tolerance of abalone
Source: Evol Appl. 2022 May 13;15(6):992–1001. doi: 10.1111/eva.13388 (PMC9234619; doi:10.1111/eva.13388)

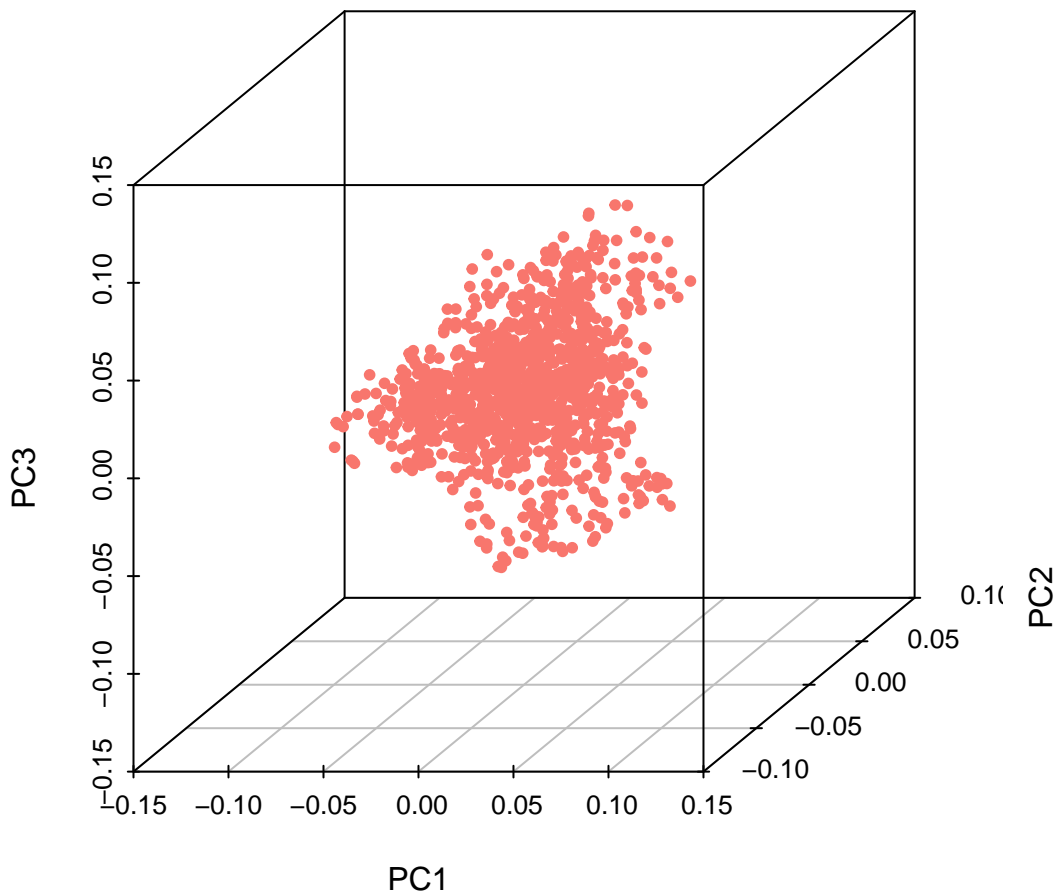

Supplement: Supplementary file 1 — Fig S1 [file EVA-15-992-s001.pdf]
